# Rationale and design of a randomized trial on the impact of aldosterone antagonism on cardiac structure and function in diabetic cardiomyopathy

**DOI:** 10.1186/1475-2840-12-139

**Published:** 2013-10-01

**Authors:** Melissa Leung, Vincent W Wong, Stephane Heritier, Anastasia S Mihailidou, Dominic Y Leung

**Affiliations:** 1 Department of Cardiology, Liverpool Hospital, Locked Bag 7103, Liverpool BC, NSW 1871, Australia; 2 Diabetes and Endocrine Service, Liverpool Hospital, Sydney, Australia; 3 University of New South Wales, Sydney, Australia; 4 The George Institute, University of Sydney, Sydney, Australia; 5 Cardiovascular & Hormonal Research Laboratory, Department of Cardiology, Kolling Institute of Medical Research, Royal North Shore Hospital & University of Sydney, Sydney, Australia

**Keywords:** Diabetes, Heart failure, Left ventricular function, Echocardiography, Collagen, Aldosterone antagonism

## Abstract

Development of a cardiomyopathy in diabetes mellitus is independent of traditional risk factors, with no clinical trials targeting specific therapeutic interventions. Myocardial fibrosis is one of the key mechanisms and aldosterone is a key mediator of myocardial fibrosis. We propose that aldosterone antagonism will improve cardiac function. We aim to evaluate the efficacy of selective aldosterone receptor antagonism with eplerenone added to optimal medical treatment in improving cardiac structure and function in diabetic cardiomyopathy. We will randomize 130 patients with type 2 diabetes mellitus, stable metabolic control and impaired left ventricular (LV) systolic or diastolic function, to either eplerenone (target dose 50mg) or matching placebo, in addition to optimal medical therapy for 12 months. The primary endpoints are changes in LV systolic and diastolic function, measured by echocardiographic 2-dimensional speckle tracking strain and strain rate and tissue Doppler imaging. The secondary endpoints include changes in echocardiographic markers and plasma biomarkers of collagen turnover; left atrial dimensions and function, incidence of atrial fibrillation and changes in exercise capacity and dyspnea score. The present study will assess whether specific aldosterone antagonism with eplerenone in addition to standard therapy will prevent progression or reverse cardiac dysfunction in diabetic cardiomyopathy using sensitive, robust and quantifiable echocardiographic measures that allow early detection of change. The study may offer a new direction in the management of this condition.

**Trial registration:**

ACTRN12610001063000

## Background

**Figure 1:**
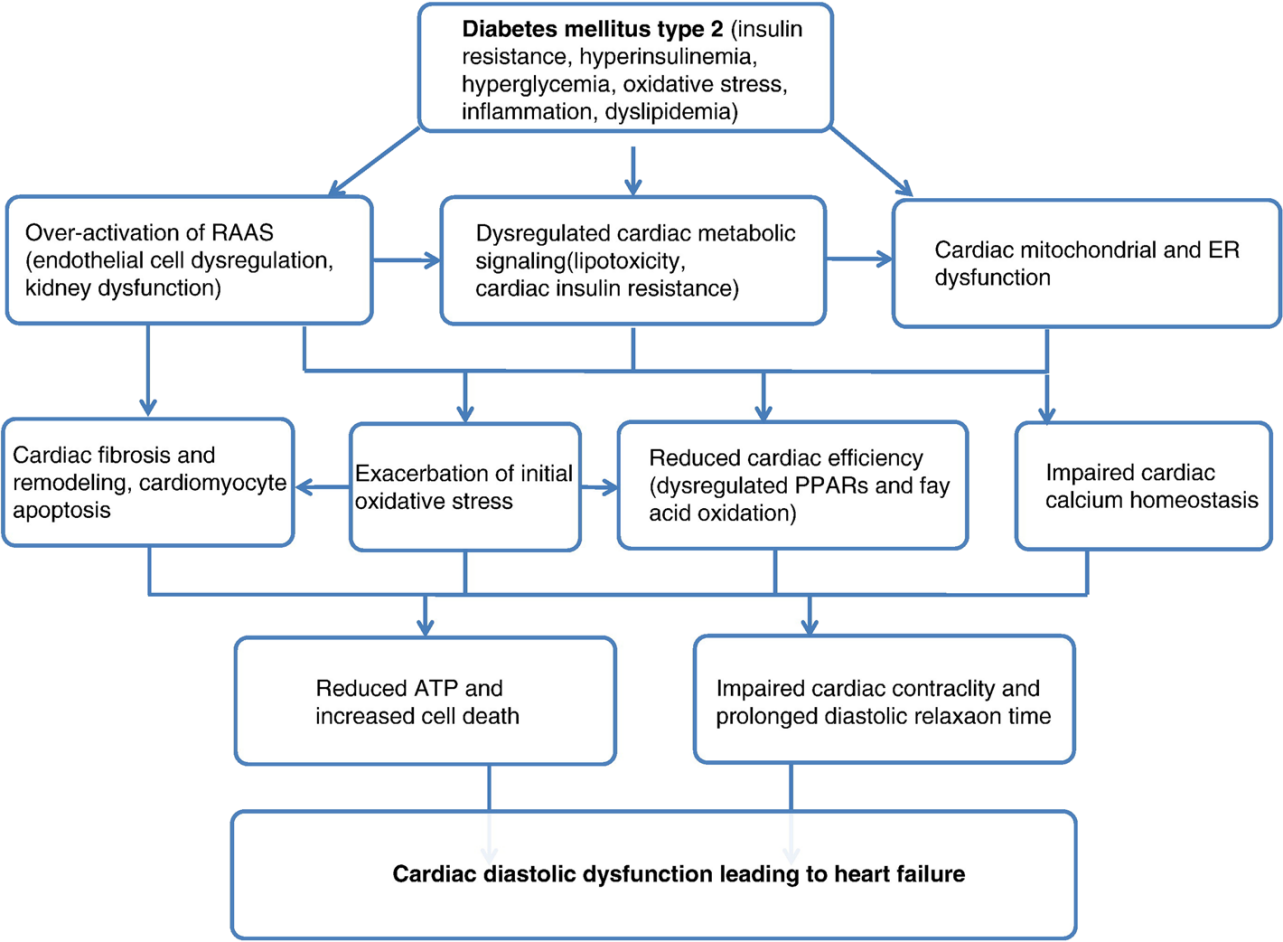
**Proposed scheme of development and progression of cardiac dysfunction as a result of the metabolic abnormalities seen in diabetes.** Legend: RAAS = renin–angiotensin II–aldosterone system, PPARs = peroxisome proliferator activator receptors, ER = endoplasmic reticulum, ATP = adenosine triphosphate. From Mandavia CH, Aroor AR, DeMarco VG, Sowers JR: Molecular and metabolic mechanisms of cardiac dysfunction in diabetes. *Life sciences* 2012, **92**:601–608 [9]. Copyright 2012 by Elsevier Inc. Reprinted with permission.

The incidence of obesity and type 2 diabetes mellitus (T2DM) are increasing towards epidemic levels in both developing and industrialized countries. Patients with T2DM are at increased risk of heart failure from diabetic cardiomyopathy [1], with the spectrum of disease ranging, in early stages, from diastolic dysfunction to overt systolic dysfunction during later stages. The pathogenesis of ventricular dysfunction in diabetic cardiomyopathy is multifactorial, and independent of traditional cardiovascular risk factors such as hypertension, hyperlipidemia and coronary artery disease (CAD) (Figure 1) [2-5]. Patients with heart failure and preserved ejection fraction, which occurs in the early stages of diabetic cardiomyopathy, experience prognosis and functional decline similar to patients with heart failure and reduced ejection fraction. Evidence based management guidelines are limited for heart failure with preserved ejection fraction. Treatment is mainly directed towards symptomatic relief with loop diuretics and treatment of associated conditions such as hypertension. Definitive treatment to halt disease progression, improve prognosis and reverse the underlying pathophysiology is still lacking.

Myocardial fibrosis has been suggested as one of the key mechanisms underlying myocardial dysfunction in T2DM [1]. Cardiac extracellular matrix (ECM) plays an active role in modulating cardiac function and undergoes extensive and continuous turnover, an important step in an injury-reparative process. Myocardial fibrosis involving predominantly types I and III collagen, the most abundant type in the heart, has been documented in biopsy studies of diabetic patients without hypertension or CAD [6,7]. Furthermore, the synthesis of type III collagen has been found to proportionally increase with hyperglycemia [8].

Overactivation of the renin angiotensin-II aldosterone system (RAAS) occurs in diabetic cardiomyopathy resulting in cardiac insulin resistance and a cascade of abnormalities mediated by angiotensin II and aldosterone (Figure 1). Aldosterone plays an important role in promoting fibrosis by stimulating fibroblast proliferation and collagen synthesis, triggering proinflammatory factors leading to activation of matrix metalloproteinases (MMPs), and increasing transforming growth factor-β (TGF-β) [9]. Aldosterone antagonism has been shown to improve symptoms and outcomes in advanced systolic heart failure and after acute myocardial infarction (AMI). Attenuation of the excessive ECM turnover by aldosterone antagonism was suggested as one of the mechanisms underlying its beneficial effects in systolic heart failure [10,11]. The beneficial effects of aldosterone antagonism on myocardial fibrosis may help to explain the impressive improvement in patient outcomes in systolic heart failure and after AMI despite only modest improvement in left ventricular (LV) ejection fraction. Aldosterone antagonism with spironolactone administered to patients with mildly symptomatic dilated cardiomyopathy improved LV diastolic function and led to regression in myocardial fibrosis on biopsy [12]. Similar improvements in LV function and measures of fibrosis have been documented in patients with hypertensive cardiomyopathy [13] and metabolic syndrome [14]. However, the efficacy and safety of aldosterone antagonism has not been previously evaluated in early stage diabetic cardiomyopathy (ranging from isolated diastolic dysfunction to systolic dysfunction with NYHA functional class I-II symptoms) where aldosterone antagonists are currently not indicated.

There is paucity of data on the specific use of aldosterone antagonists in T2DM and this is a particular group who are prone to hyporeninemic hypoaldosteronism [15]. As a consequence, there are concerns regarding hyperkalemia with the use of aldosterone antagonists, particularly in combination with angiotensin converting enzyme inhibitors (ACEI) or angiotensin receptor blockers (ARB). Evaluation of this particular problem in clinical trials has been limited to subgroup analyses. The present study will provide safety information on the use of such drugs in patients with T2DM.

Circulating biomarkers of collagen synthesis and degradation provide a means of monitoring ECM turnover and the balance between collagen synthesis and degradation [16-22]. Levels of the, procollagen type III N-terminal propeptide (PIIINP) and procollagen type I C-terminal propeptide (PICP) have been shown to have prognostic value in systolic heart failure [10,11], with aldosterone antagonism decreasing levels of these biomarkers. Moreover, clinical benefits were most predominant in those who had elevated biomarker levels [11].

Cardiac imaging modalities such as echocardiography and cardiac magnetic resonance imaging can be used to quantify myocardial interstitial fibrosis. Histologically quantified collagen accumulation has been shown to be linearly related to the magnitude of ultrasound backscatter, a marker of myocardial reflectivity, in experimental [23,24] as well as in clinical studies [25,26]. The magnitude of integrated backscatter has been shown to correlate with serum concentrations of PICP and PIIINP in patients with hypertensive heart disease [27]. Diabetic patients without hypertension, CAD, or overt heart failure demonstrate evidence of systolic dysfunction and abnormal calibrated integrated backscatter (IB). These changes are not only similar to those caused by LV hypertrophy, but are also independent and incremental to the effects of ventricular hypertrophy [28]. The potential for assessing retardation or regression of this fibrosis using ultrasound or biomarker surrogates in patients with subclinical diabetic cardiomyopathy has not been investigated. Furthermore, in contrast to the use of ejection fraction for assessing serial change in cardiac function, echocardiographic strain and strain rate imaging provides a robust, sensitive, reproducible and quantifiable measure of changes in cardiac function that precede changes in ejection fraction. Documentation of strain and strain rate measures have the potential to allow earlier detection of abnormalities as well as changes in cardiac function to effect earlier intervention strategies before the development of advanced heart disease.

The aim of the current study is to investigate whether treatment with eplerenone, a specific aldosterone antagonist, for 12 months, in addition to ACEI or ARB, would improve myocardial structural and functional abnormalities in patients with diabetic cardiomyopathy without advanced heart failure or severe LV systolic dysfunction.

### Research design and methods

**Figure 2:**
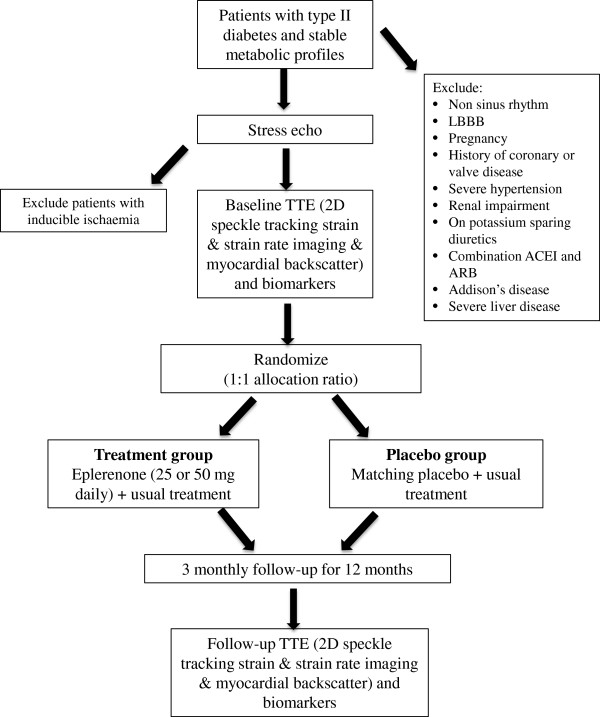
**Flow chart showing study design.** Legend: TTE: transthoracic echo; LBBB: left bundle branch block; ACEI: angiotensin converting enzyme inhibitors; ARB: angiotensin receptor blockers; 2D: 2 dimensional.

This study was designed as a prospective, investigator-initiated, multi-centre, randomized, double blind, placebo controlled study to evaluate the impact of treatment with eplerenone for 12 months on myocardial structure and function in patients with T2DM and LV diastolic or systolic dysfunction, in the absence of class III-IV symptoms with severe LV systolic dysfunction. The study was conducted in accordance with the NHMRC guidelines and was approved by Sydney Southwest Area Health Service Human Research Ethics committee. Figure 2 shows the study design and protocol.

## Patients

### Inclusion criteria

Eligible subjects include male and female adults that meet the following criteria:

 1) An established diagnosis of T2DM of any duration and severity.

 2) Presence of LV diastolic or systolic dysfunction.

 3) Stable metabolic control (target blood pressure ≤135/75 mmHg, lipids (total cholesterol ≤4.0 mmol/L, LDL ≤ 2.0 mmol/L, TG ≤ 1.0 mmol/L), blood glucose levels (HbA1c ≤ 8.0%)) such that minimal or no dose changes of other medications, such as ACEI, ARB, lipid lowering and blood glucose lowering medications are anticipated during the course of the study.

 4) NYHA functional class I or II.

 5) Treatment with optimal target or maximal tolerated dose of ACEI or ARB and ß-blocker, unless contraindicated.

 6) Serum potassium ≤5.0 mmol/L within 1 week prior to randomization.

 7) Estimated glomerular filtration rate (eGFR) > 30 ml/min/1.73m^2^.

 8) Subjects who received previous treatment with aldosterone antagonists for more than 7 consecutive days must meet the following additional criteria:

a. No history of clinically significant hyperkalemia or renal impairment during previous aldosterone antagonist treatment.

a. Aldosterone antagonist treatment has been discontinued for at least 3 months prior to randomization.

### Exclusion criteria

Subjects are not eligible for enrolment if they present any of the following criteria:

 1) Non-sinus rhythm.

 2) Pregnancy.

 3) Severe heart failure (class III-IV, LV ejection fraction <35%).

 4) Significant CAD (patients who have not undergone recent coronary angiography will have significant CAD excluded by stress echocardiography).

Further exclusion criteria pertain specifically to the use of aldosterone antagonists, and include:

 1) Serum potassium >5.0 mmol/L.

 2) eGFR < 30 ml/min/1.73m^2^.

 3) Concomitant potassium sparing diuretic.

 4) Combination of ACEI with ARB.

 5) Addison’s disease.

 6) Acute renal insufficiency.

 7) Severe hepatic insufficiency.

### Screening and randomization

Preliminary screening for trial eligibility is based on review of medical records by clinical trials staff at or before the baseline visit. After written informed consent, eligible subjects are randomly assigned by the clinical trial pharmacist to treatment with eplerenone or matching placebo. Randomization is by permuted blocks and is conducted via an electronic data management system.

### Echocardiography protocol

All patients will undergo resting echocardiography with strain and strain rate imaging for evaluation of LV and left atrial (LA) function. Myocardial calibrated IB will be performed on grey scale 2-dimensional (2D) images to assess the presence and extent of myocardial fibrosis.

#### Two-dimensional and Doppler echocardiography

All transthoracic echocardiograms will be performed with Vivid E9 (GE Medical Systems, Milwaukee, Wisconsin). All standard echo and Doppler parameters of LV systolic and diastolic function including tissue Doppler (TDI) will be measured. Maximum, pre-P, and minimum LA volumes will be measured using Simpson’s biplane method of discs.

#### Strain and strain rate imaging

**Figure 3:**
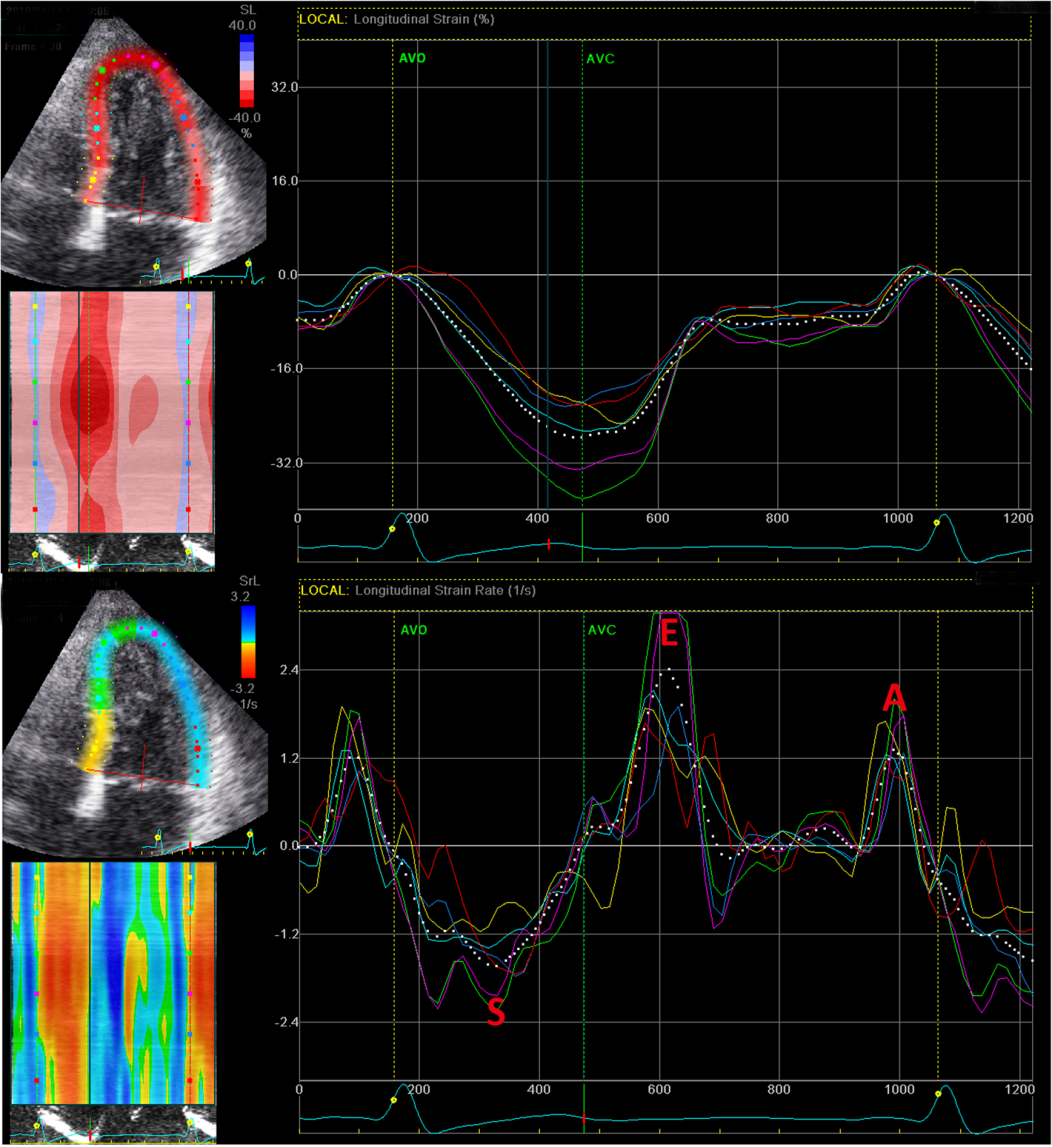
**Longitudinal strain and strain rate imaging by 2D speckle tracking for the apical 4-chamber view.** The top panel is the strain throughout one cardiac cycle for each of the color coded LV segments (mean strain shown by dotted white line). In this example, the mean peak strain is about 27% which occurs during LV ejection, defined as the interval between aortic valve opening (AVO) and closure (AVC). The bottom panel shows the corresponding LV strain rate. Note the negative strain rate in systole (S) and the positive strain rates (E during early diastole, and A during late diastole).

Longitudinal strain and strain rates of the left ventricle (Figure 3) will be obtained using 2D speckle tracking. Images of the left ventricle will be acquired in the apical 4-, 2-chamber and long axis views using highest possible frame rates.

**Figure 4:**
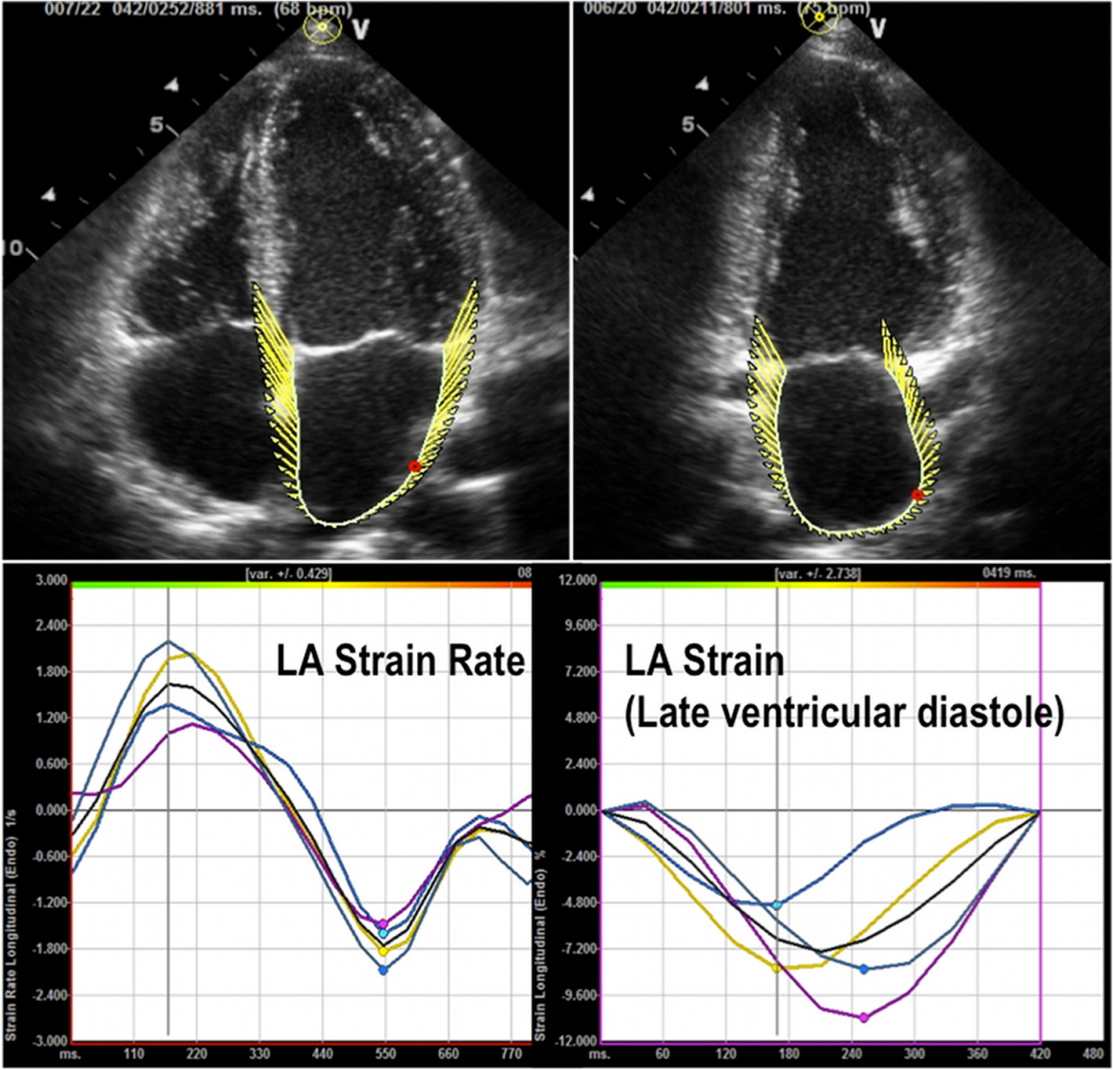
**Velocity vector imaging for left atrial strain and strain rate in the apical 4-chamber *(left images) *and apical 2-chamber *(right images) *views.** The right lower image is an example of LA strain during atrial systole and the left lower image is an example of LA strain rate curves. In the left lower image, the black line represents the global LA strain rate during LV systole, early and late diastole. The colored lines represent the strain rates of the two annular and mid segments of the LA. The right lower image shows the global and segmental LA strain during atrial systole (late ventricular diastole).

Left atrial longitudinal strain and strain rate will be similarly obtained in 2 annular and 2 mid segments of the apical 4- and 2-chamber views using velocity vector imaging (VVI; syngo Velocity Vector Imaging technology, Siemens Medical Solutions, Ultrasound Division, Mountain View, California) (Figure 4). VVI will be used for LA strain analysis due to the thin-walled nature of the LA wall, and the improved tracking with VVI compared to 2D-speckle tracking for the left atrium.

#### Stress echocardiography

Stress echocardiography will be performed according to standard protocols. Images will be acquired at rest and peak exercise. Two-dimensional images of the left ventricle will be obtained in the apical (4 chamber, 2 chamber, long axis) and short axis (midventricular and apical) views.

#### Calibrated integrated backscatter

**Figure 5:**
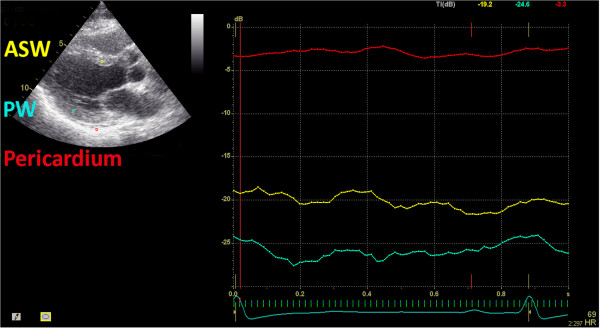
**Assessment of left ventricular fibrosis by calibrated integrated backscatter (IB) in the parasternal long axis view.** A 9 × 9 pixel sample volume is placed in the mid-myocardium of the basal anteroseptum, posterior wall, and pericardium, and is tracked throughout the cardiac cycle. In this example, calibrated IB for the anteroseptum is obtained by subtracting average pericardial IB intensity (−3.3 dB) from average myocardial IB intensity of the septum (−19.2 dB), which results in a calibrated IB for the septum of −15.9 dB.

The IB curves (Figure 5) will be extracted in the parasternal long-axis view, using standard software (Echopac, GE Vingmed). Measurements will be obtained by placing a 9x9-pixel sample volume in the subendocardial basal anteroseptum, posterior wall, and pericardium in end-diastole in the parasternal long axis view. The position of the sample volume will be checked and adjusted in each frame to keep the sample volume within the same region during the whole cardiac cycle. Calibrated IB is obtained by subtracting average pericardial IB intensity from average myocardial IB intensity of the anteroseptum or posterior wall.

### Biomarker assays

Biomarker levels will be taken prior to commencement of the study medication, as well as after 12 months treatment. Peripheral venous blood will be obtained between 8am and 9am after 15 minutes of supine rest. Serum P1NP and P3NP will be measured by radioimmunoassay (Orion Diagnostica, Espoo, Finland); serum P1CP will be measured by enzyme-linked immunoadsorbent assay (Takara Bio, Shiga, Japan); plasma MMP-1 and MMP-9 will be measured by immunoassay (Millipore Corp, Missouri, USA); plasma TIMP-1 levels will be measured by enzyme-linked immunoadsorbent assay (RayBiotech Inc, Georgia, USA); serum TGF-ß1 will be measured using an enzyme-linked immunoadsorbent assay (R&D Systems, Minneapolis, Minnesota); and serum direct renin and aldosterone levels will be quantified by radioimmunoassay (Renin III Generation RIA kit, Cisbio Bioassays, Bagnols-Sur-Cèze, France; and Coat-A-Count Aldosterone, Siemens Healthcare Diagnostics, Eschborn, Germany; respectively). For quality control and validation purposes, all assays will be performed twice.

### Functional tests

A 6-minute walk test will be performed and Medical Research Council (MRC) dyspnea score recorded at randomization and after 12 months of therapy.

### Dosing regimen

Eplerenone and placebo tablets and packaging are identical in appearance and are manufactured as 25mg tablets by Pfizer (Pfizer, Inc, New York, USA). Subjects are commenced on 25mg eplerenone or matching placebo (1 × 25mg tablet) orally once daily, and may have their dose up-titrated to 50mg (2 × 25mg tablet) after 4 weeks in the absence of worsening renal function or hyperkalemia. In accordance with guidelines for management of heart failure [29,30], serum electrolytes and renal function will be tested 1 and 4 weeks after dose increase/commencement, then 3-monthly thereafter. Study drug dose adjustment or dose maintenance is guided by safety considerations, in particular changes in serum potassium and renal function, which are monitored at each visit. Patients will continue all other usual medical treatments, whilst blood pressure, serum cholesterol, hemoglobin A1c (HbA1c) and sugar diary will be monitored at 3-monthly intervals to ensure ongoing optimal metabolic control. Blood pressure and heart rate will be measured in the sitting position with a newly calibrated semiautomatic oscillometric device with a digital readout (Omron T9, Omron Healthcare, Melbourne, Australia). The subjects will be seated in a quiet room and blood pressure measurements started after a 10–15min rest period. Measurements will be performed in the same arm each time and with a cuff size appropriate to the patient’s arm. The cuff will be kept at the heart level, and the arm will be supported at the time of the measurement. Three measurements will be made at least 15 seconds apart, and the averages recorded. Visits will take place at the same time of the day, usually in the morning, Treatment will be maintained and monitored for 12 months, when repeat cardiac imaging, functional testing and biomarker analysis will be performed.

### Endpoints

The primary endpoints are changes in global mean peak LV systolic strain and strain rate, peak early diastolic myocardial tissue Doppler velocities at the basal septal LV wall (e’), the ratio of mitral inflow early diastolic velocity to peak early diastolic mitral annular velocity (E/e’), LV calibrated IB, LV mass index and LA strain and strain rate at ventricular systole and early and late ventricular diastole, and LA phasic volumes. Secondary endpoints include changes in collagen turnover measured as changes in serum levels of procollagen type I aminoterminal peptide (PINP), PIIINP, PICP, matrix metalloproteinases 1 and 9 (MMP-1, MMP-9), tissue inhibitor of metalloproteinase-1 (TIMP-1), renin, and aldosterone levels). Other secondary endpoint measurements include MRC dyspnea score, exercise tolerance, as measured by 6-minute walk test distance and incidence of atrial fibrillation.

Serious adverse events (SAE) including life-threatening hyperkalemia (>7mmol/L), acute renal failure, hypotension and death will be monitored.

### Hypotheses

 1. Aldosterone antagonism with eplerenone in diabetic cardiomyopathy leads to an improvement in LV systolic and diastolic function, measured by 2D speckle tracking strain and strain rate imaging.

 2. Myocardial fibrosis, as measured by echocardiographic calibrated IB and serum collagen biomarkers is reduced by eplerenone.

 3. Eplerenone improves LA function and phasic volumes.

 4. Changes in LV and LA function with eplerenone are correlated with changes in the degree of myocardial fibrosis.

### Statistical considerations

#### Sample size determination

At the time of study design and commencement, there were no studies that examined the impact of aldosterone antagonism with eplerenone on cardiac function as measured by strain and strain rate imaging and cardiac fibrosis in patients with diabetic cardiomyopathy. Therefore, sample size calculation was based on previous studies that examined the effects of spironolactone on strain and strain rate in patients with diastolic heart failure from dilated and hypertensive cardiomyopathies, changes in myocardial calibrated IB in these patients, as well as differences in IB between patients with early diabetic cardiomyopathy and normal controls [12,13,28,31]. The change in IB between baseline and 12 month echocardiograms will be measured in both placebo and eplerenone arms (ΔIB). A sample size of 65 patients per treatment group (130 patients in total) will achieve 80% power to detect a difference in ΔIB of −4.5 dB (SD = 7) with two-sided α = 0.05 using a two-sample t-test. This includes a 10% loss to follow-up rate for clinic visits and repeat echocardiograms, as well as a 15% non-compliance rate with eplerenone or placebo. A power of at least 80% was also obtained for peak longitudinal systolic strain and strain rate, and early diastolic strain rate using similar calculations.

Since commencement of this study, Kosmala and colleagues [14] examined the effects of spironolactone versus placebo on cardiac function in patients with metabolic syndrome and found improvements in longitudinal strain and strain rate over a 6-month period, but no significant difference in ΔIB. Sample size recalculation has shown that the intended sample size of 130 patients in the present study provides adequate power to detect a difference over the 12 month time period in LV strain and strain rate.

These sample size calculations are based on a two-sample t-test and are therefore conservative. As analysis of covariance (ANCOVA) adjusted for the baseline measurement will be the method of analysis, the power will be higher or the minimal detectable effect smaller for the same power target. The gain in efficiency is linked to the correlation (r) between the baseline and on-treatment measurements which is currently unknown for the endpoints involved, but expected to be high [32].

#### Trial oversight

A data safety and monitoring board (DSMB) comprising of personnel with sufficient cardiological, endocrinological and statistical experience will be formed to review the interim results and oversee the conduct of the trial. The DSMB and ethics committee will review all unblinded data on clinical endpoint events and determine whether events meet pre-specified criteria. The DSMB will monitor safety and efficacy of the trial and periodically assess whether the trial should continue.

#### Interim analysis

Interim analyses for safety and efficacy are incorporated into this trial. The Haybittle-Peto rule with two interim efficacy analyses detailed in a statistical analysis plan are planned after approximately 30% and 60% of the patients have been followed up at 12 months. Early termination for the study will be recommended for evidence of overwhelming benefit (2-sided p < 0.003 favouring eplerenone) or substantive harm (2-sided p < 0.01 against eplerenone).

#### Statistical analysis

The intention-to-treat principle will be applied to the analysis. Baseline and demographic characteristics will also be summarized by treatment group. All changes from baseline, including the primary and secondary end-points will be analysed by means of ANCOVA, adjusted for the baseline measurement.

Descriptive statistics will be provided for safety data. The number of patients reporting any SAE, the occurrence of specific SAEs and discontinuation due to SAEs will be tabulated. The effect of treatment on the number of patients experiencing at least one SAE will be tested using a χ^2^-test or Fisher’s exact test if the number of events is small. All hypotheses are two-sided. A p-value of <0.05 is considered statistically significant.

## Discussion

There are features of this trial design that deserve particular emphasis:

● Patients of all symptomatology and degrees of myocardial abnormalities ranging from grade 1–3 diastolic dysfunction to mild to moderate systolic dysfunction are enrolled. Including patients with more severe grades of baseline diastolic or systolic dysfunction may allow greater improvement on eplerenone. Furthermore, patients with milder degrees of systolic/diastolic dysfunction randomized to placebo may experience disease progression during the course of the study.

● Blood pressure independent effects of eplerenone will be examined by recruiting those with stable blood pressure control at baseline, and blood pressure will be continually monitored to ensure stability throughout the study. Furthermore, the dose of eplerenone will not exceed 50mg daily, minimizing the antihypertensive effects.

● The impact of aldosterone antagonism on myocardial fibrosis will be measured both by cardiac imaging and serum levels of biomarkers. Furthermore, attempts will be made to correlate the changes in myocardial function with changes in the degree of myocardial fibrosis both on imaging as well as changes in biomarker levels.

● In comparison to previous studies examining biomarkers of fibrosis, it is assumed that the beneficial effects of aldosterone antagonism come from more effective suppression of aldosterone, yet few have definitively shown that this beneficial effect is present despite a persistently elevated renin and aldosterone level after treatment. We will measure both renin and aldosterone level before and after treatment in our study.

## Summary

Diabetic cardiomyopathy is an increasingly prevalent disorder for which there is currently no specific therapeutic intervention. This trial, when successfully completed, will advance our understanding of diabetic cardiomyopathy and the underlying mechanisms. This may provide a new direction in the management of diabetes; the early treatment by eplerenone may prevent and reverse the progression of atrial and ventricular dysfunction and myocardial fibrosis to irreversible structural and symptomatic cardiac disease. Thus, the proposed research will have a favourable impact in turning the focus of treatment of this disease from secondary prevention and heart failure symptom management into an interventional approach at the preclinical stage. This may result in a reduction in morbidity and mortality for patients with T2DM, with improved outcomes and consequent substantial health cost savings for the community.

## Abbreviations

2D: 2-dimensional; ACEI: Angiotensin converting enzyme inhibitors; AMI: Acute myocardial infarction; ARB: Angiotensin receptor blockers; CAD: Coronary artery disease; IB: Integrated backscatter; DSMB: Data safety and monitoring board; e’: Peak early diastolic velocity; E/e’: Ratio of mitral inflow early diastolic velocity to peak early diastolic mitral annular velocity; HbA1c: Glycosylated hemoglobin A1c; LA: Left atrial; LV: Left ventricular; MMPs: Matrix metalloproteinases; MMP-1: Matrix metalloproteinase 1; MMP-9: Matrix metalloproteinase 9; NYHA: New York Heart Association functional class; PICP: Procollagen type I carboxy-terminal propeptide; PIIINP: Procollagen type III amino-terminal propeptide; PINP: Procollagen type I aminoterminal peptide; RAAS: Renin angiotension-II aldosterone system; TGF-β: Transforming growth factor-β; TIMP-1: Tissue inhibitor of metalloproteinase 1; T2DM: Type 2 diabetes mellitus.

## Competing interests

Pfizer Inc. provided eplerenone and placebo tablets for this trial. However, Pfizer has no role in study design, data collection, analysis, interpretation of data, or manuscript preparation. ML has previously received a CardioVascular Lipid research grant support from the sponsor for an unrelated study but has no other financial conflicts.

## Authors’ contributions

ML jointly conceived the study with DL, performed literature review, designed and implemented the trial, obtained ethics approval, have been involved in the recruitment of subjects, review of trial patients, data collection, and preparation of the manuscript. ML and SH conducted statistical trial planning, SH generated the random allocation sequence and concealment mechanism, reviewed and edited the manuscript, VW contributed towards recruitment and review of subjects, reviewed and edited the manuscript, AM reviewed and edited the manuscript. All authors read and approved the final manuscript.
